# Supplementary material for: A Cdk1 phosphomimic mutant of MCAK impairs microtubule end recognition
Source: PeerJ. 2017 Dec 6;5:e4034. doi: 10.7717/peerj.4034 (PMC5723132; doi:10.7717/peerj.4034)
Supplement: Table S1 — Values for the microtubule lattice residence times, microtubule lattice and end dissociation constants and microtubule association constants for WT-MCAK and the phosphomimic mutant T537E. [file peerj-05-4034-s001.docx]

Supplementary Table S1

| MCAK variant | WT | T537E |
| --- | --- | --- |
|  | **(**s**)** (mean ± SEM) | |
| Lattice residence time | 0.48 ± 0.02  (n = 526) | 0.42 ± 0.01  (n = 622) |
|  |  |  |
|  | **(s^-1^)** (mean ± SD) | |
| *k_off_* (MT lattice) | 2.90 ± 0.16 | 2.64 ± 0.19 |
| *k_off_* (MT end) | 0.98 ± 0.06 | 2.30 ± 0.40 |
|  |  |  |
|  | **(s^-1^)** (mean ± SD) | |
| *k_on_* (MT) | 0.59 ± 0.28  (n = 3) | 0.48 ± 0.06  (n = 3) |
