# Supplementary figures and images for: A Cdk1 phosphomimic mutant of MCAK impairs microtubule end recognition
Source: PeerJ. 2017 Dec 6;5:e4034. doi: 10.7717/peerj.4034 (PMC5723132; doi:10.7717/peerj.4034)

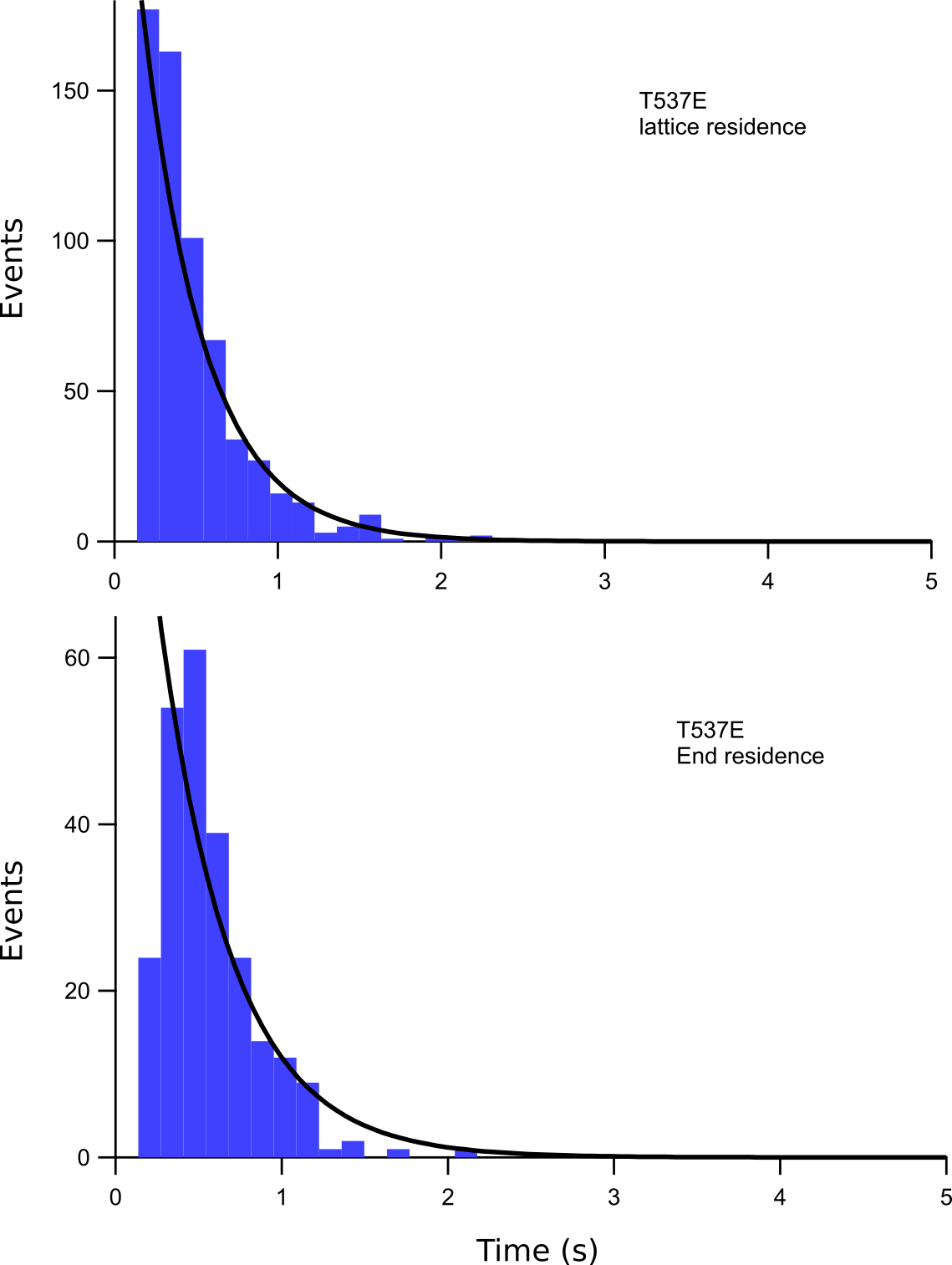

Supplement: Figure S1 — Histograms showing the number of microtubule lattice and microtubule end interaction events of a particular residence time for T537E. The fits of these data to a single exponential function are shown in black. The microtubule lattice and microtubule end dissociation constants obtained from these fits are listed in Supplementary Table S1. [file peerj-05-4034-s002.png]

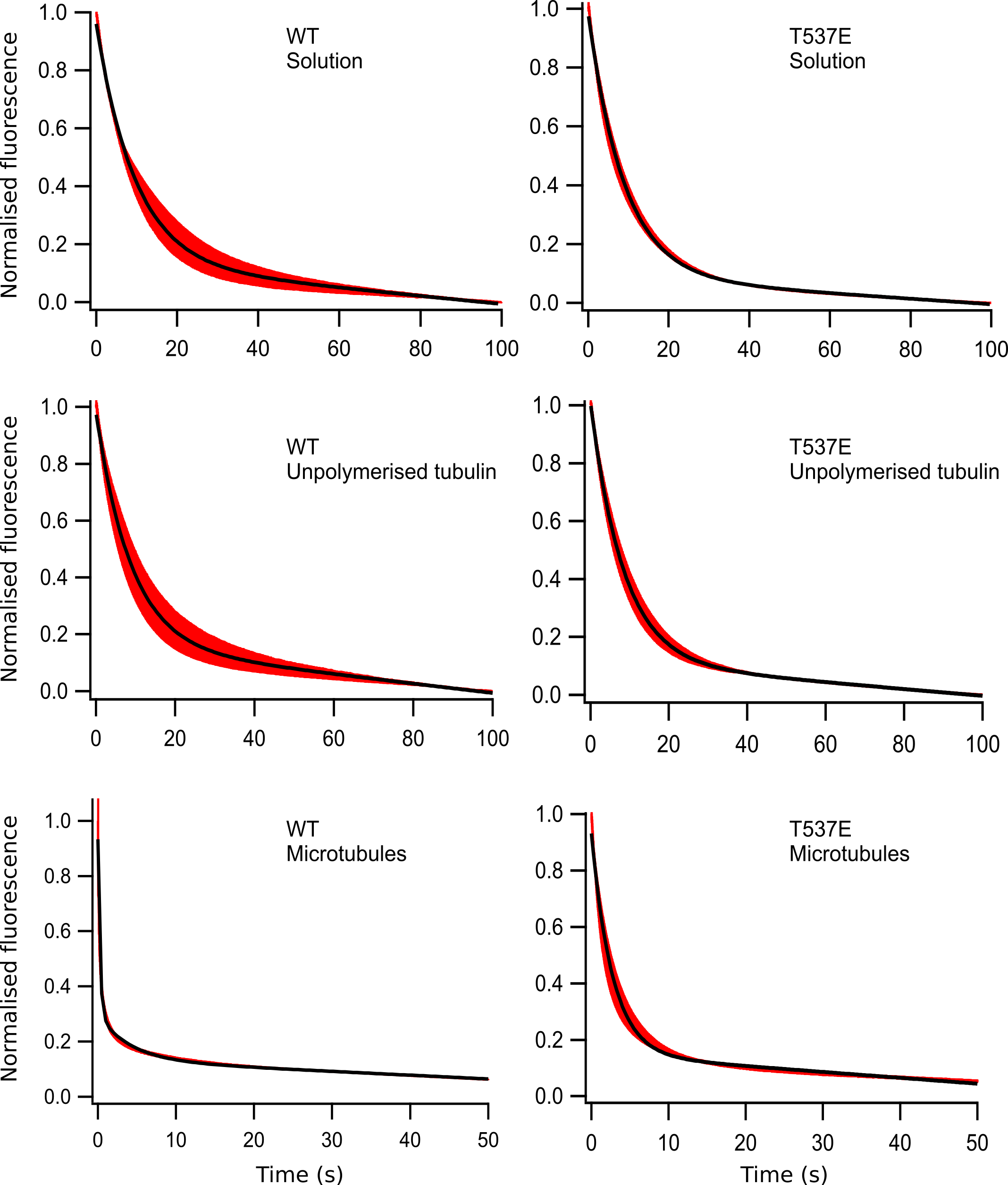

Supplement: Figure S2 — Fluorescence transients (red) resulting from the dissociation of mantADP from WT-MCAK or the phosphomimic mutant T537E. The average of 3 data sets ±the standard deviation is shown. The data has been normalised to allow comparison. The fit to each transient of either a single or double exponential function plus a line of constant negative slope to account for photobleaching of the mant group is shown in black. The data for WT-MCAK and T537E both in solution and in the presence of unpolymerized tubulin and T537E in the presence of microtubules are fit to a single exponential. Rate constants were determined from fitting the raw data and were as follows: 0.102 ±0.013 s−1 and 0.114 ±0.013 s−1 in solution and 0.114 ±0.023 s−1 and 0.120 ±0.019 s−1 in the presence of unpolymerized tubulin for WT and T537E, respectively. The rate constant for T537E in the presence of microtubules was 0.341 ±0.051 s−1. The data for WT-MCAK in the presence of microtubules were fit to a double exponential with rate constants of k1 = 4.11 ± 0.41s−1 and k2 = 0.341 ± 0.051s−1. [file peerj-05-4034-s003.png]
